# Naloxone Use in Novel Potent Opioid and Fentanyl Overdoses in Emergency Department Patients

**DOI:** 10.1001/jamanetworkopen.2023.31264

**Published:** 2023-08-29

**Authors:** Alexandra Amaducci, Kim Aldy, Sharan L. Campleman, Shao Li, Alison Meyn, Stephanie Abston, Rachel E. Culbreth, Alex Krotulski, Barry Logan, Paul Wax, Jeffrey Brent, Alex F. Manini

**Affiliations:** 1 Lehigh Valley Health Network-USF Morsani College of Medicine, Allentown, Pennsylvania; 2 American College of Medical Toxicology, Phoenix, Arizona; 3 Baylor University Medical Center, Dallas, Texas; 4 Center for Forensic Science Research and Education at the Fredric Rieders Family Foundation, Willow Grove, Pennsylvania; 5 NMS Labs, Horsham, Pennsylvania; 6 University of Texas Southwestern Medical Center, Dallas; 7 University of Colorado School of Medicine, Aurora; 8 NYC Health and Hospitals, Elmhurst, New York; 9 Icahn School of Medicine at Mount Sinai, New York, New York

## Abstract

**Importance:**

Synthetic opioids, such as the fentanyl analogue and nitazene drug class, are among the fastest growing types of opioids being detected in patients in the emergency department (ED) with illicit opioid overdose (OD). However, clinical outcomes from OD of novel potent opioids (NPOs), specifically nitazenes, are unknown aside from small case series.

**Objective:**

To determine naloxone administration and clinical sequelae of patients who were in the ED with NPO overdose compared with fentanyl OD.

**Design, Setting, and Participants:**

This is a cohort study subgroup analysis of adults admitted to the ED and tested positive for NPOs among in the ongoing nationwide ToxIC Fentalog cohort study from 2020 to 2022. Patients who were in the ED with a presumed acute opioid OD and residual blood samples were included, and those testing positive for NPOs were analyzed. Patients were included in this analysis if their confirmatory testing was positive for an NPO analyte, such as brorphine, isotonitazene, metonitazene, and/or N-piperidinyl etonitazene. A comparison group included patients that were positive for fentanyl and devoid of any other analytes on toxicologic analysis.

**Exposures:**

Patients were exposed to NPOs, including brorphine, isotonitazene, metonitazene and/or N-piperidinyl etonitazene.

**Main Outcomes and Measures:**

The primary outcome was the total number of naloxone doses and total cumulative naloxone dose administered as part of routine clinical care following the OD. Naloxone requirements and clinical sequelae of NPO-positive patients were compared with those testing positive for fentanyl only.

**Results:**

During the study period, 2298 patients were screened, of whom 717 met inclusion criteria, 537 had complete laboratory testing data, with 11 (2.0%) positive for only fentanyl and 9 (1.7%) positive for NPOs (brorphine, isotonitazene, metonitazene, or N-piperidinyl etonitazene). The age range of patients was aged 20 to 57 years (4 males [44.4%] and 5 females [55.6%]). The NPO group received a statistically significantly higher mean (SD) number of naloxone boluses in-hospital (1.33 [1.50]) compared with the fentanyl group (0.36 [0.92]) (*P* = .02), which corresponded to a moderately large effect size (Cohen *d* = 0.78). Metonitazene overdose was associated with cardiac arrest and more naloxone doses overall. Metonitazene cases had a mean (SD) number of 3.0 (0) naloxone doses, and 2 of 2 patients (100%) with metonitazene overdoses were administered cardiopulmonary resuscitation.

**Conclusions and Relevance:**

In this cohort study of patients admitted to the ED with confirmed opioid overdose testing positive for NPOs, in-hospital naloxone dosing was high compared with patients who tested positive for fentanyl alone. Further study is warranted to confirm these preliminary associations.

## Key Points

**Question:**

What are the naloxone requirements and clinical sequelae of emergency department patients with novel potent opioid (NPO) overdose exposures?

**Findings:**

In this cohort study of 537 patients, all patients with NPO overdose presented with opioid overdose symptoms and received multiple doses of naloxone. Compared with fentanyl overdose, patients with NPO overdose had a higher number of naloxone doses administered in-hospital; metonitazene overdose was associated with cardiac arrest and more naloxone doses overall.

**Meaning:**

These findings suggest that NPOs may have a higher potency than fentanyl due to the observed naloxone administration in the clinical setting of overdose.

## Teaser

This cohort study examines naloxone administration among US adults admitted to the emergency department (ED) with a novel potent opioid overdose compared with fentanyl overdose.

## Introduction

Novel potent opioids (NPOs) are novel nonfentanyl opioids in the illicit opioid supply. Synthetic opioids are one of the fastest growing classes of opioids being detected in patients in the emergency department (ED) with opioid overdose (OD).^1^

A subclass of synthetic opioids referred to as nitazenes contain a 2-benzylbenzimidazole structure that has μ-opioid agonism. Isotonitazene, metonitazene, and N-piperidinyl etonitazene are NPOs with a piperidine benzimidazolone structure. Brorphine is a nonnitazene NPO that is a full μ-opioid receptor agonist with a structure similar to fentanyl. NPOs possess high potency at the μ-opioid receptor and an understudied propensity for adverse health effects. Nitazenes are structurally unrelated to fentanyl, but have been found to be up to 1000-fold more potent than morphine.^1^

The exact motivation to produce nitazenes and brorphine are unclear. The increased regulation of fentanyl and fentanyl analogues throughout the last decade may have led to a change in the chemical precursors required for clandestine laboratory production that were not yet regulated.^1^ This change in chemical precursors may have led to these newer and more potent opioids.

This study focuses on the clinical implications of brorphine, isotonitazene, metonitazene, and N-piperidinyl etonitazene following nonfatal OD. Though the in vitro potency of some NPOs has been evaluated,^1^ there is a substantial gap in the literature of the clinical characteristics of nonfatal opioid ODs involving NPOs. Specifically, naloxone requirements and OD severity for NPOs are unknown. Therefore, this is the first study to our knowledge to describe the naloxone requirements and clinical sequelae of patients presenting to the ED with confirmed NPO drug OD.

## Methods

This cohort study follows the Strengthening the Reporting of Observational Studies in Epidemiology (STROBE) reporting guideline. This study was reviewed and approved with a waiver of patient consent by the WIRB-Copernicus Group institutional review board. Informed consent was waived because deidentified patient data were used.

This is a subgroup analysis of the Toxicology Investigators Consortium (ToxIC) Fentalog study, an ongoing, national multicenter study to investigate nonfatal opioid OD.^2,3^ Patients in the ED who presented to 1 of 10 participating sites with a presumed opioid OD were eligible and included if waste or discarded blood specimens were available after being drawn as part of routine clinical care. Exclusion criteria were children (ie, younger than 18 years), persons who were incarcerated, and those presenting with concern for burns or physical trauma. Patients were included in this subgroup analysis if their confirmatory testing was positive for any of the following NPOs (ie, NPO group): brorphine, isotonitazene, metonitazene, and/or N-piperidinyl etonitazene. The control group for the subgroup analysis included those testing positive for only fentanyl and without presence of other opioids, other drug classes, or any adulterants (ie, fentanyl group). Deidentified clinical information was collected by medical record review from the patient’s electronic medical record by medical toxicology physician site investigators at each site, and entered into a central REDCap database, including: demographics, past medical history, prior substance use, naloxone administration (including prehospital doses if known), in-hospital clinical course, vital signs, and disposition. Data regarding race and ethnicity were obtained from the patient’s medical record (self-identified or registration observed during routine hospital intake) with categories based on US Office of Management and Budget standards, which included American Indian and Alaska Native, Asian, Black and African, Caucasian, Native Hawaiian or other Pacific Islander, White, other, and unknown.^4^ Race and ethnicity were included in this study to control for differences in NPO exposure that may be attributed to race and ethnicity. No long-term follow-up was performed. Discarded blood specimens that were drawn as part of routine clinical care were collected, deidentified, linked to the clinical database, and shipped to a central laboratory for toxicology analysis. To preserve confidentiality, these biological samples were deidentified with a code linking the sample to the corresponding REDCap registry entry. Samples were analyzed by the Center for Forensic Science Research and Education for qualitative toxicologic confirmation performed by liquid chromatography quadrupole time-of-flight mass spectrometry to detect the presence of more than 1000 substances. Substances tested for include classic illicit drugs, novel psychoactive substances, adulterants, and their metabolites. The scope of testing is updated each year as the substances in this dynamic illicit drug market evolve frequently. The lower limit of quantitation is determined to be 0.1 ng/mL for all analytes. Turnaround time to allow for batching of samples does not allow for toxicology results to be available in real time. All analyses were performed blinded to clinical data and outcomes. This method has been validated successfully according to a Scientific Working Group in Forensic Toxicology (SWGTOX) compliant approach and in a previously published analysis of novel synthetic opioids in blood, serum or plasma, and urine in forensic casework.^5^ In addition, analyses using this method for novel synthetic opioids from the Fentalog Study Group have been published in Clinical Toxicology,^3^ and in other forensic casework publications.^6,7^

The primary outcome was total naloxone administration. This outcome was measured, based on medical record review of the in-hospital and prehospital medical record, in 2 ways: (1) the number of naloxone bolus doses (not including infusion) in-hospital and prehospital; and, (2) total cumulative dose (in mg). Furthermore, the total cumulative naloxone dose was analyzed in 3 ways as a sensitivity analysis—total dose including infusions, total dose excluding infusions, and total dose excluding infusions and the first dose. Prehospital doses were assumed 2 mg intranasal when not otherwise specified. Resolution of signs of the clinical opioid toxidrome (ie, coma, miosis, and respiratory depression) was determined based on medical record review, whenever possible, by each study site investigator.

Secondary outcomes were severe clinical sequelae following OD, based on medical record review of the in-hospital and prehospital medical record. These included (1) cardiopulmonary resuscitation (CPR; yes or no), (2) orotracheal intubation (yes or no), and (3) severe cardiovascular outcomes (after 4 hours). Severe cardiovascular outcomes were defined as the presence of at least 1 of the following: ventricular dysrhythmias (ventricular tachycardia or fibrillation or torsades des pointes), prolonged QRS (>120 ms), prolonged QTc (>500 ms), atrioventricular block greater than first degree, cardiac arrest (loss of pulse), and/or elevated troponin (yes or no, based on each hospital’s definition as per its own troponin assay).

### Statistical Analysis

Statistical analysis consisted of computing descriptive statistics (eg, absolute and relative frequencies) on demographics and clinical characteristics between the NPO group and the corresponding fentanyl group. Primary and secondary outcomes were examined for statistically significant differences using bivariate statistical tests (eg, Fisher Exact tests for categorical variables and Mann-Whitney U tests for continuous variables). Statistical significance testing was set a priori at *P* < .05. All analyses were conducted in R version 4.2.1 (R Project for Statistical Computing).

## Results

Between September 21, 2020, and September 27, 2022, 2298 patients were screened, 717 met inclusion criteria, and 537 patients had complete laboratory testing data (Figure). Among the 717 included cases, 223 patients (31.1%) were female, 477 (66.5%) were male, 2 were transgender (0.3%), and 15 (2.1%) were missing sex. The mean (SD) age was 42.4 (14.5) years. Of these, 9 patients were positive for either brorphine, isotonitazene, metonitazene, or N-piperidinyl etonitazene (NPO group), and 11 were positive for fentanyl without other opioids (fentanyl group). The overall age range of patients was aged 20 to 57 years; the demographics and differences between the NPO and Fentanyl groups are outlined in Table 1. The mean age for the NPO group was nearly 15 years older compared with the Fentanyl Group.

**Figure.**
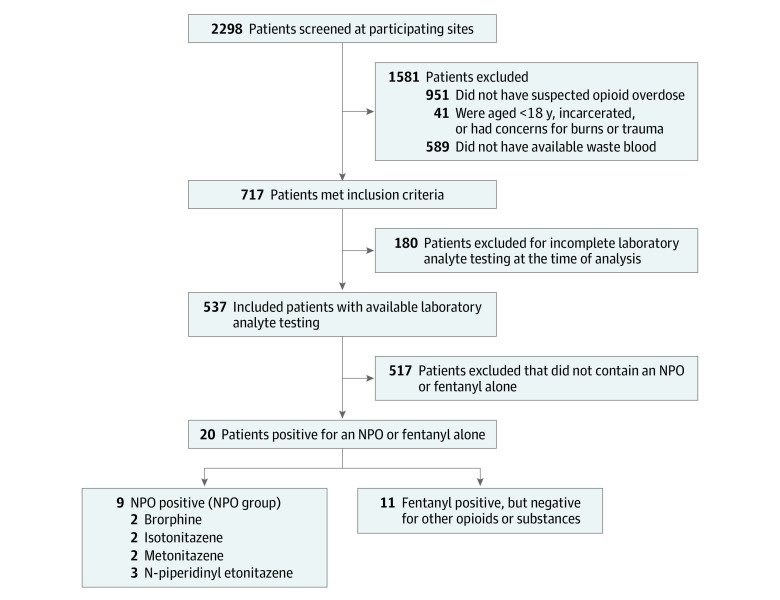
Patient Eligibility and Groups

**Table 1.** Demographic Characteristics for Each NPO Compared With Fentanyl Only Group

Characteristic	Patients, No. (%)					
	Fentanyl only group (n = 11)	Brorphine (n = 2)	Isotonitazene (n = 2)	Metonitazene (n = 2)	N-piperidinyl etonitazene (n = 3)	NPO group (n = 9)^a^
Age, mean (SD)	28.4 (5.2)	46.5 (14.9)	45.5 (6.4)	34.50 (20.5)	45.33 (11.2)	43.22 (11.9)
Sex						
Male	10 (90.9)	1 (50.0)	1 (50.0)	0	2 (66.7)	4 (44.4)
Female	1 (9.1)	1 (50.0)	1 (50.0)	2 (100.0)	1 (33.3)	5 (55.6)
Race or ethnicity						
Black	2 (18.2)	2 (100.0)	0	0	0	2 (22.2)
Hispanic	0	0	0	0	0	0
Other^b^	1 (9.1)	0	2 (100.0)	1 (50.0)	2 (66.7)	5 (55.6)
White	8 (72.7)	0	0	1 (50.0)	1 (33.3)	2 (22.2)

^a^ NPO group includes brorphine, isotonitazene, metonitazene, and N-piperidinyl etonitazene.

^b^ The other race or ethnicity category includes American Indian or Alaskan Native, Asian, Native Hawaiian or Pacific Islander, mixed race, and uncertain categories.

All 9 patients in the NPO group (100%) presented with an opioid toxidrome and received naloxone (Table 2). Two patients (22%) were positive for brorphine, 2 patients (22%) were positive for isotonitazene, 2 patients (22%) were positive for metonitazene, and 3 patients (33%) were positive for N-piperidinyl etonitazene. The 2 patients who tested positive for brorphine reported using what was believed to be a parenteral opioid, such as heroin or fentanyl. Both patients had a depressed level of consciousness (DLOC) and respiratory depression, which was reversed with 1 dose of naloxone. Isotonitazene was detected in 2 patients; both had DLOC and received 2 naloxone doses to reverse symptoms. The 2 patients who were found to have metonitazene presented in cardiac arrest; 1 patient died despite a total of 6 mg of naloxone in 3 separate doses, and the other patient survived after receiving a total of 10 mg of naloxone in 3 doses. N-piperidinyl etonitazene was detected in 3 patients (33%) (and has been previously reported).^3^ The first patient presented with low oxygen saturation and DLOC and had resolution of signs after 3 total doses of naloxone (2 mg, 0.04 mg, and 0.04 mg). The second patient had respiratory depression and DLOC and had resolution of signs after a 1-time dose of 8 mg of naloxone. The third patient had respiratory depression and DLOC but had resolution of signs after 5 bolus doses of naloxone (0.04 mg, 0.04 mg, 0.2 mg, 0.4 mg, and 0.04 mg) followed by an infusion of naloxone (2.4 mg).

**Table 2.** Case Narratives and Naloxone Dosing for Each Patient in NPO Group (N = 9)

Case	Age and sex	State	Naloxone treatment and indication	Naloxone response	Reported opioid exposure in medical record	Laboratory NPO and opioid analytes	Level of care	Hospital length of stay, h
1	30s Male	Missouri	0.16 mg IV for respiratory depression and low SpO_2_	Resolution of signs	Fentanyl or fentanyl analogue	Brorphine, fentanyl	ED only	10
2	50s Female	Missouri	2 mg IM for respiratory depression and DLOC	Resolution of signs	Heroin, fentanyl or fentanyl analogue	Brorphine, heroin, fentanyl, acetyl fentanyl	ED only	8
3	40s Female	Michigan	2 unknown dose IN for DLOC	Resolution of signs	Heroin	Isotonitazene, heroin, para-fluorofentanyl, fentanyl	ED only	10
4	50s Male	Michigan	4 mg IN and 0.2 mg IV for DLOC	Resolution of signs	Heroin	Isotonitazene, heroin, fentanyl	Floor	67
5	20s Female	California	2 mg IM and 2 doses 2 mg IV for cardiac arrest	No response	Fentanyl or fentanyl analogue	Metonitazene, fentanyl	ICU	867
6	40s Female	Michigan	2 doses 4 mg IN for cardiac arrest, 2 mg IN for respiratory depression	Resolution of signs	Heroin, fentanyl or fentanyl analogue	Metonitazene, fentanyl	ED observation unit	21
7	30s Male	New Jersey	4 doses 0.04 mg IV and 0.2 mg IV for respiratory depression and DLOC, 2.4 mg IV total via infusion	Resolution of signs	Heroin	N-piperidinyl etonitazene, heroin, para-fluorofentanyl, fentanyl	ED observation unit	202
8	40s Female	New Jersey	8 mg IN for respiratory depression and DLOC	Resolution of signs	Opioid unspecified	N-piperidinyl etonitazene	ED only	26
9	50s Male	New Jersey	2 mg IN for low oxygen and DLOC, 2 0.04 mg IV for low SpO_2_	Resolution of signs	Opioid unspecified	N-piperidinyl etonitazene, fentanyl	ICU	141

Abbreviations: DLOC, depressed level of consciousness; ED, emergency department; ICU, intensive care unit; IM, intramuscular; IN, intranasal; IV, intravenous; SpO_2_, oxygen saturation.

Descriptive analysis of both primary and secondary outcomes are demonstrated in Table 3 for the NPO group compared with the fentanyl group. Six of the NPO group received 2 or more doses of naloxone (66.6%) compared with the fentanyl group (36.4% received 2 or more doses of naloxone). The NPO group received a statistically significantly higher number of naloxone boluses in-hospital compared with the fentanyl group (mean [SD], 1.33 [1.50] vs 0.36 [0.92], respectively; *P* = .02). This difference corresponds to a moderate-to-large effect size (Cohen *d* = 0.78).

**Table 3.** Naloxone Administration and Clinical Characteristics for Individual and Combined NPOs Compared With Fentanyl Only Group

Naloxone administration^a^	Patients, No. (%)						NPO vs fentanyl only, *P* value
	Brorphine (n = 2)	Isotonitazene (n = 2)	Metonitazene (n = 2)	N-piperidinyl etonitazene (n = 3)	NPO group (n = 9)^b^	Fentanyl only group (n = 11)	
Naloxone administration							
No or unknown	0	0	0	0	0	1 (9.1)	
Yes	2 (100)	2 (100)	2 (100)	3 (100)	9 (100)	10 (90.9)	>.99
Prehospital naloxone							
Yes	0	2 (100)	2 (100)	2 (66.7)	6 (66.7)	10 (90.9)	
No	2 (100)	0	0	1 (33.3)	3 (33.3)	1 (9.1)	.28
Prehospital total No. of boluses, mean (SD)	0 (0)	2 (0)	2.00 (0)	0.67 (0.58)	0.78 (0.83)	1.28 (0.79)	.56
In-hospital naloxone							
Yes	2 (100)	1 (50.0)	2 (100)	2 (66.7)	7 (77.8)	3 (27.3)	
No	0	1 (50.0)	0	1 (33.3)	2 (22.2)	8 (72.7)	.07
In-hospital total No. of boluses, mean (SD)	1.00 (0)	0.50 (0.71)	1.00 (0)	2.33 (2.52)	1.33 (1.50)	0.36 (0.92)	.02
Total No. of naloxone boluses, mean (SD)	1.00 (0)	2.00 (0)	3.00 (0)	3.00 (2.00)	2.33 (1.32)	1.64 (1.36)	.17
Total cumulative naloxone dose in mg (including first dose and infusions), mean (SD)	1.08 (1.30)	4.10 (0.14)	8.00 (2.83)	4.40 (3.16)	4.40 (3.12)	6.41 (6.58)	.79
Total cumulative naloxone dose in mg (excluding first dose and infusions), mean (SD)	0	1.10 (1.28)	5.00 (1.41)	0.25 (0.37)	1.44 (2.18)	1.05 (1.85)	.37
Total cumulative naloxone dose in mg (including first dose and excluding infusions), mean (SD)	1.08 (1.30)	4.10 (0.14)	8.00 (2.83)	3.60 (3.87)	4.13 (3.34)	4.68 (3.00)	.79
Infusion required							
No	2 (100.0)	2 (100.0)	2 (100.0)	2 (66.7)	8 (88.9)	10 (90.9%)	
Yes	0	0	0	1 (33.3)	1 (11.1)	1 (9.1%)	>.99
Secondary outcomes							
CPR initiated							
No	2 (100.0)	2 (100.0)	0	3 (100.0)	7 (77.8)	7 (63.6)	
Yes	0	0	2 (100.0)	0	2 (22.2)	4 (36.4)	.64
Intubation							
No	2 (100.0)	2 (100.0)	1 (50.0)	3 (100.0)	8 (88.9)	8 (72.7)	
Yes	0	0	1 (50.0)	0	1 (11.1)	3 (27.3)	.59
Cardiovascular effects after 4 h							
No	2 (100.0)	2 (100.0)	1 (50.0)	3 (100.0)	8 (88.9)	10 (90.9)	
Yes	0	0	1 (50.0)	0	1 (11.1)	1 (9.1)	>.99
Death							
No	2 (100.0)	2 (100.0)	1 (50.0)	3 (100.0)	8 (88.9)	10 (90.9)	
Yes	0	0	1 (50.0)	0	1 (11.1)	1 (9.1)	>.99

Abbreviations: CPR, cardiopulmonary resuscitation; NPO, novel potent opioid.

^a^ Total naloxone dosage was calculated among all individuals. For individuals where it was unknown if naloxone was given, a 0 was assigned for that specific dose. Patients who had a missing dose for intranasal naloxone were imputed at 2 mg.

^b^ NPO group includes brorphine, isotonitazene, metonitazene, and N-piperidinyl etonitazene.

The mean (SD) total cumulative naloxone dose administered between groups was similar (NPO, 4.40 (3.12) mg vs fentanyl, 6.41 (6.58) mg). A significant outlier existed in the fentanyl group who received a total of 18 mg of naloxone infusion and 6.12 mg naloxone dose through bolus administration, resulting in a cumulative amount of 24.12 mg of naloxone. However, the groups were similar in total naloxone dosing in mg overall. The mean (SD) total cumulative naloxone dose administered (excluding the first dose and excluding infusions) was also comparable between the NPO group (1.44 [2.18] mg) and the fentanyl group (1.05 [1.85] mg). Lastly, the total cumulative naloxone dose, including the first dose but excluding infusions, was 4.68 mg for the fentanyl group and 4.13 mg for the NPO group.

Intubation was performed on 1 of 2 patients using metonitazene (50.0%), whereas 3 of 11 patients (27.3%) who used only fentanyl tients were intubated. CPR was initiated in 4 of 11 patients (36.4%) in the fentanyl only group, but 2 of 2 patients (100%) of the metonitazene group received CPR. Overall, the prevalence of secondary outcomes in the fentanyl group compared with the NPO group was similar.

Table 4 documents the demographic and clinical characteristics among individual NPO groups compared with other NPOs. The metonitazene group received the highest mean (SD) cumulative amount of naloxone (8.00 [2.83] mg) followed by N-piperidinyl etonitazene (4.40 [3.16] mg), isotonitazene (4.10 [0.14] mg), and brorphine (1.08 [1.30] mg).

**Table 4.** Demographics and Clinical Outcomes Comparing Individual NPO Groups

Characteristics	Patients, No. (%)							
	Brorphine vs other NPO		Isotonitazene vs other NPO		Metonitazene vs other NPO		N-piperidinyl etonitazene vs other NPO	
	Other NPO (n = 7)	Brorphine (n = 2)	Other NPO (n = 7)	Isotonitazene (n = 2)	Other NPO (n = 7)	Metonitazene (n = 2)	Other NPO (n = 6)	N-piperidinyl etonitazene (n = 3)
Demographics								
Age, Mean (SD) years	48.0 (12.5)	46.5 (10.5)	48.0 (17.5)	45.5 (4.5)	4.0 (14.0)	34.5 (20.5)	45.0 (12.5)	45.3 (11.2)
Sex								
Male	3 (42.9)	1 (50.0)	3 (42.9)	1 (50.0)	4 (57.1)	0	2 (33.3)	2 (66.7)
Female	4 (57.1)	1 (50.0)	4 (57.1)	1 (50.0)	3 (42.9)	2 (100)	4 (66.7)	1 (33.3)
Race or ethnicity								
Black	0	2 (100)	2 (28.6)	0	2 (28.6)	0	2 (33.3)	0
Other^a^	5 (71.4)	0	3 (42.9)	2 (100)	4 (57.1)	1 (50.0)	3 (50.0)	2 (66.7)
White	2 (28.6)	0	2 (28.6)	0	1 (14.3)	1 (50.0)	1 (16.7)	1 (33.3)
Naloxone administration^b^								
Prehospital naloxone								
Yes	6 (86.7)	0	4 (57.1)	2 (100)	4 (57.1)	2 (100)	4 (66.7)	2 (66.7)
No	1 (14.3)	2 (100)	3 (42.9)	0	3 (42.9)	0	2 (33.3)	1 (33.3)
Prehospital total No. of boluses, mean (SD)	1.3 (0.8)	0	0.9 (0.9)	1.5 (0.7)	0.7 (0.8)	2.0 (0)	1.2 (1.0)	0.7 (0.6)
In-hospital naloxone								
Yes	5 (71.4)	2 (100)	6 (85.7)	1 (50.0)	5 (71.4)	0	5 (83.3)	2 (66.7)
No	2 (28.6)	0	1 (14.5)	1 (50.0)	2 (28.6)	2 (100)	1 (16.7)	1 (33.3)
In-hospital total No. of boluses, mean (SD)	1.4 (1.7)	1.00 (0)	1.6 (1.6)	0.5 (0.7)	1.4 (1.7)	1.0 (0)	0.8 (0.4)	2.3 (2.5)
Total No. of naloxone boluses, mean (SD)	2.7 (1.3)	1.0 (0)	2.4 (1.5)	2.0 (0)	2.1 (1.5)	3.0 (0)	2.0 (1.0)	3.0 (2.0)
Total cumulative naloxone dose in mg (including first dose and infusions), mean (SD)	5.3 (2.8)	1.1 (1.3)	4.5 (3.6)	4.1 (0.1)	3.4 (2.5)	8.0 (2.8)	4.4 (3.4)	4.4 (3.2)
Total cumulative naloxone dose in mg (excluding first dose and infusions), mean (SD)	1.9 (2.3)	NA	1.5 (2.5)	1.1 (1.3)	0.4 (0.7)	5.0 (1.4)	2.0 (2.5)	0.3 (0.4)
Total cumulative naloxone dose in mg (including first dose and excluding infusions), mean (SD)	5.0 (3.3)	1.1 (1.3)	4.1 (3.9)	4.1 (0.1)	3.0 (2.7)	8.0 (2.8)	4.4 (3.4)	3.6 (3.9)
Infusion required								
No	6 (85.7)	2 (100)	6 (85.7)	2 (100)	6 (85.7)	2 (100)	6 (100)	2 (66.7)
Yes	1 (14.3)	0	1 (14.3)	0	1 (14.3)	0	0	1 (33.3)
Clinical outcomes								
CPR initiated								
No	5 (71.4)	2 (100)	5 (71.4)	2 (28.6)	7 (100)	0	4 (66.7)	3 (100)
Yes	2 (28.6)	0	2 (28.6)	0	0	2 (100)	2 (33.3)	0
Intubation								
No	6 (85.7)	2 (100)	6 (85.7)	2 (100)	7 (100)	1 (50.0)	5 (83.3)	3 (100)
Yes	1 (14.3)	0	1 (14.3)	0	0	1 (50.0)	1 (16.7)	0
Cardiovascular effects after 4 h								
No	6 (85.7)	2 (100)	6 (85.7)	2 (100)	7 (100)	1 (50.0)	5 (83.3)	3 (100)
Yes	1 (14.3)	0	1 (14.3)	0	0	1 (50.0)	1 (16.7)	0

Abbreviations: CPR, cardiopulmonary resuscitation; NPO, novel potent opioid.

^a^ Other race or ethnicity category includes American Indian or Alaskan Native, Asian, Native Hawaiian or Pacific Islander, mixed race, and unknown race categories.

^b^ Total naloxone dosage was calculated among all patients. For patients who did not receive naloxone, 0 mg was imputed. Patients who had a missing dose for intranasal naloxone were imputed at 2 mg.

## Discussion

This study is the first, to our knowledge, to document the clinical sequelae and naloxone administration for patients who were in the ED following confirmed NPO drug OD. The NPO group was administered a statistically significantly higher number of in-hospital naloxone boluses compared with the fentanyl group, which corresponded to a moderately large effect size. While these findings were based on limited sample sizes, we detected a large effect size for the association between increased naloxone doses and NPO overdose. The majority of patients with ODs that involved NPO received 2 or more doses of naloxone, whereas most of the patients who OD from fentanyl only received 1 dose of naloxone. While this study was statistically underpowered to detect differences in naloxone administration in total cumulative dosage and clinical sequelae between patients with NPO and fentanyl only OD, this study provides important preliminary data on NPOs to inform clinicians and patients of the severity of ODs involving NPOs. Furthermore, this preliminary data underscores the urgent need to study NPOs in a larger, future cohort. These data suggest that NPOs may have higher potency than fentanyl and by extension heroin.

NPOs, such as nitazenes and brorphine, are relatively new to the illicit opioid supply but may have significant clinical implications based on the findings of the present study. The nitazene drug class is structurally unrelated to fentanyl but has been found to be up to 1000-fold more potent than morphine.^1^ Isotonitazene and brorphine are 2 of the NPOs that emerged as novel psychoactive substances (NPS) in 2019 and 2020, respectively. Brorphine was initially created in 2018 and first noted on the recreational drug market in 2019.^8^ Nitazenes and brorphine have consistently demonstrated stronger in vitro μ-opioid receptor activation than fentanyl^1^ but until now translation of these findings to the clinical setting has been difficult.

In general, naloxone dosing was high in both groups studied, which may have been influenced by the prehospital administration of 2 mg naloxone intranasally in most regions studied. In the present study, most patients in the NPO group required multiple doses of naloxone. However, the fentanyl only group received almost 50% more naloxone than the NPO groups. Thus, while the present study was underpowered to calculate statistically significant differences in cumulative naloxone administration, true population differences may exist and require further study.

NPOs have higher μ-opioid receptor activation than morphine and fentanyl, which has previously been demonstrated by an in vitro study which pharmacologically evaluated 10 nitazenes using 2 cell-based β-arrestin2/mini-Gi recruitment assays monitoring μ-opioid receptor activation.^1^ In addition, there appear to be differences within each drug in the nitazene class. In the present study, metonitazene appears to have the most severe clinical toxicity given that both patients in which metonitazene was detected presented in cardiac arrest, and 1 patient died.

Findings from the present study can begin to inform emergency care clinicians about naloxone administration for NPO overdose. Our data have public health implications and may provide insight for emergency care clinicians and bystanders who administer naloxone. Specifically, the need for higher numbers of doses of naloxone in the NPO group, as well as the association between metonitazene OD and cardiac arrest, pose a public health threat. Clinicians should be aware of these opioids in the drug supply so they are adequately prepared to care for these patients and anticipate needing to use multiple doses of naloxone. In addition, to date there has been a lack of bystander education on repeat naloxone dosing.^9^ This is an opportunity for public health awareness and to educate those providing bystander naloxone and patients using illicit opioids about the higher potential for OD and death, and that repeat doses of naloxone may be needed to reverse NPO overdose. Similarly, this is an opportunity for education among patients with substance use disorder as a public health outreach to educate the potential additional adverse effects with the rise of NPOs in the NPS drug supply.^10,11^

Many patients may believe they are using heroin; however, the number of deaths caused by heroin is declining, while the overall number of opioid-related deaths is increasing.^12^ The ongoing ToxIC Fentalog study group has reported via public health alerts a rise in fentanyl and fentanyl-analogue OD in patients who were in the ED across the country,^2^ but now the addition of nitazenes presents an additional layer of complexity that may not be well known by patients and clinicians alike.

While metonitazene appears to be the most clinically severe OD in this subgroup analysis, patients that had brorphine, isotonitazene, and N-piperidinyl etonitazene in their serum all presented with opioid OD toxidromes and most required multiple doses of naloxone. For example, 1 patient with N-piperidinyl etonitazene received 5 bolus doses of naloxone followed by an infusion before resolution of signs. Another patient with N-piperidinyl etonitazene requiring an initial dose of naloxone of 8 mg. Prior case reports have suggested that patients with NPO overdose are at higher risk for fatality,^10,11^ and future research should examine statistically significant differences in cumulative naloxone dose and other severe clinical outcomes. Regardless, emergency clinicians should be aware of NPOs and be prepared to treat accordingly.

### Limitations

This study has limitations. Data collection for this study was limited to abstraction from the medical record rather than in-person interviews. Toxicology analysis was qualitative only, as technical limitations (primarily the low volume of discarded blood specimens) precluded a more sophisticated quantitative analysis. Also, many patients who were screened were excluded due to the unavailability of waste specimens; this likely contributed to a patient population with a relatively high clinical OD severity. Due to sample size limitations, we were unable to delineate specific signs and symptoms specific to NPOs. Additionally, many patients were exposed to NPOs in combination with fentanyl; thus toxicity may have been due to the fentanyl rather than the NPO. Furthermore, because of the small sample size of the NPO group (9 patients) and the comparison group of fentanyl only (11 patients), statistical testing was only conducted on primary and secondary outcomes. Future studies should incorporate a larger sample of cases with NPO overdose to determine the differences between NPO and fentanyl only OD with regards to cumulative naloxone dose and other clinical characteristics. Because of the small sample size, the number of naloxone doses may also be related to geographical differences rather than the total number of naloxone doses required to reverse the OD. Additionally, this study only consists of patients presenting to the ED at 1 of the participating hospitals in the ToxIC network, and thus may not be generalizable to all clinical settings. This data did not include prehospital decedents.

## Conclusions

In this cohort study of patients in the ED with confirmed opioid overdose testing positive for NPOs, in-hospital naloxone dosing was high compared with fentanyl alone. Additionally, patients with metonitazene OD experienced cardiac arrest and death at a higher rate than those involving other substances. Further study is warranted to confirm these preliminary associations.

## Notes

Supplement 1.
Toxicology Investigators Consortium Fentalog Study Group

Supplement 2.
Data Sharing Statement

## References

[1] Vandeputte MM, Van Uytfanghe K, Layle NK, St Germaine DM, Iula DM, Stove CP. Synthesis, chemical characterization, and μ-opioid receptor activity assessment of the emerging group of “nitazene” 2-benzylbenzimidazole synthetic opioids. ACS Chem Neurosci. 2021;12(7):1241-1251. doi:10.1021/acschemneuro.1c00064 33759494

[2] Center for Forensic Science Research and Education. ToxIC Fentalog Study Group—quarterly NPS report. Accessed March 27, 2023. https://www.cfsre.org/nps-discovery/clinical-reports

[3] Calello DP, Aldy K, Jefri M, ; ToxIC Fentalog Study Group. Identification of a novel opioid, *N*-piperidinyl etonitazene (etonitazepipne), in patients with suspected opioid overdose. Clin Toxicol (Phila). 2022;60(9):1067-1069. doi:10.1080/15563650.2022.2084406 35708103 PMC9815205

[4] Revisions to the standards for the classification of federal data on race and ethnicity. Federal Register. Accessed July 27, 2023. https://www.govinfo.gov/content/pkg/FR-1997-10-30/pdf/97-28653.pdf

[5] Moody MT, Diaz S, Shah P, Papsun D, Logan BK. Analysis of fentanyl analogs and novel synthetic opioids in blood, serum/plasma, and urine in forensic casework. Drug Test Anal. 2018;10(9):1358-1367. doi:10.1002/dta.2393 29633785

[6] Mohr ALA, Friscia M, Papsun D, Kacinko SL, Buzby D, Logan BK. Analysis of novel synthetic opioids U-47700, U-50488 and furanyl fentanyl by LC-MS/MS in postmortem casework. J Anal Toxicol. 2016;40(9):709-717. doi:10.1093/jat/bkw086 27590036

[7] Papsun D, Krywanczyk A, Vose JC, Bundock EA, Logan BK. Analysis of MT-45, a novel synthetic opioid, in human whole blood by LC-MS-MS and its identification in a drug-related death. J Anal Toxicol. 2016;40(4):313-317. doi:10.1093/jat/bkw012 27091064

[8] Vandeputte MM, Krotulski AJ, Papsun DM, Logan BK, Stove CP. The rise and fall of isotonitazene and brorphine: two recent stars in the synthetic opioid firmament. J Anal Toxicol. 2021;46(2):115-121. doi:10.1093/jat/bkab082 34233349

[9] Kim HK, Connors NJ, Mazer-Amirshahi ME. The role of take-home naloxone in the epidemic of opioid overdose involving illicitly manufactured fentanyl and its analogs. Expert Opin Drug Saf. 2019;18(6):465-475. doi:10.1080/14740338.2019.1613372 31033357

[10] Montanari E, Madeo G, Pichini S, Busardò FP, Carlier J. Acute intoxications and fatalities associated with benzimidazole opioid (nitazene analog) use: a systematic review. Ther Drug Monit. 2022;44(4):494-510. doi:10.1097/FTD.0000000000000970 35149665

[11] Roberts A, Korona-Bailey J, Mukhopadhyay S. Notes from the field: nitazene-related deaths - Tennessee, 2019-2021. MMWR Morb Mortal Wkly Rep. 2022;71(37):1196-1197. doi:10.15585/mmwr.mm7137a5 36107790 PMC9484803

[12] Drug Overdose Death Rates. National Institute on Drug Abuse. Accessed March 3, 2023. https://nida.nih.gov/research-topics/trends-statistics/overdose-death-rates

