# Supplementary material for: Naloxone Use in Novel Potent Opioid and Fentanyl Overdoses in Emergency Department Patients
Source: JAMA Netw Open. 2023 Aug 29;6(8):e2331264. doi: 10.1001/jamanetworkopen.2023.31264 (PMC10466160; doi:10.1001/jamanetworkopen.2023.31264)
Supplement: Supplement 1. — Toxicology Investigators Consortium Fentalog Study Group [file jamanetwopen-e2331264-s001.pdf]

Supplemental Online Content: Nonauthor Collaborators
*First name, last name, and suffix (if applicable) are required and will appear in PubMed.

*Group Name(s): ToxIC Fentalog Study Group								
***First Name and Middle Initial(s)**	***Last Name**	***Suffix (eg, Jr, III)**	Academic Degrees	Institution	Location (city, state/province, country)	Role or Contribution, eg, chair, principal investigator	Group (if more than 1 Group listed in the byline) and/or Subgroup (eg, Steering Committee)	
Jennie	Buchanan		MD	Denver Health Medical Center	Denver, CO, USA	Site Investigator	ToxIC Fentalog Study Group	
Diane	Calello		MD	Rutgers New Jersey School of Medicine	Newark, NJ, USA	Site Investigator	ToxIC Fentalog Study Group	
Joseph	Carpenter		MD	Grady Memorial Hospital	Atlanta, GA, USA	Site Investigator	ToxIC Fentalog Study Group	
Jennifer	Carey		MD	University of Massachusetts Memorial Medical Center	Worcester, MA, USA	Site Investigator	ToxIC Fentalog Study Group	
Robert	Hendrickson		MD	Oregon Health & Science University Hospital	Portland, OR, USA	Site Investigator	ToxIC Fentalog Study Group	
Bryan	Judge		MD	Corewell Health	Grand Rapids, MI, USA	Site Investigator	ToxIC Fentalog Study Group	
Michael	Levine		MD	University of California Los Angeles	Los Angeles, CA, USA	Site Investigator	ToxIC Fentalog Study Group	
Chris	Meaden		MD	Rutgers University Hospital	Newark, NJ, USA	Site Investigator	ToxIC Fentalog Study Group	
Evan	Schwarz		MD	Washington University	St. Louis, MO, USA	Site Investigator	ToxIC Fentalog Study Group	
Joshua	Shulman		MD	University of Pittsburgh Medical Center	Pittsburgh, PA, USA	Site Investigator	ToxIC Fentalog Study Group	
