# Supplementary material for: Naloxone Use in Novel Potent Opioid and Fentanyl Overdoses in Emergency Department Patients
Source: JAMA Netw Open. 2023 Aug 29;6(8):e2331264. doi: 10.1001/jamanetworkopen.2023.31264 (PMC10466160; doi:10.1001/jamanetworkopen.2023.31264)
Supplement: Supplement 2. — Data Sharing Statement [file jamanetwopen-e2331264-s002.pdf]

Data Sharing Statement

Data
Data available: No

Additional Information
Explanation for why data not available: Data will be made available on a case-specific basis by requests made to the PI.
